# PROTOCOL: Health Visiting Interventions With 0–5 Year Olds and Their Families: An Evidence and Gap Map

**DOI:** 10.1002/cl2.70078

**Published:** 2025-12-23

**Authors:** Michael Fanner, Jane Barlow, Sarah Cowley, Karen Whittaker, Fiona Campbell, Anita Schrader McMillan, Mary Malone, Helen Critcher

**Affiliations:** ^1^ Department of Social Policy and Intervention University of Oxford Oxford UK; ^2^ Florence Nightingale Faculty of Nursing, Midwifery and Palliative Care, King's College London London UK; ^3^ Institute of Health Visiting London UK; ^4^ Population Health Sciences Institute Newcastle University Newcastle Upon Tyne UK; ^5^ Parent Advisor UK

## 1 Background

### 1.1 Introduction

#### 1.1.1 The Problem, Condition or Issue

Health visiting became a universal, statutory public health service in the United Kingdom (UK) in the first half of the twentieth century to address key public health issues (i.e., inequalities in health) and improve the health of families (Dingwall 1977). Since then, awareness of the importance of the early years in terms of the reduction of such health inequalities has led to a focus on the delivery of universal services in a proportionate way, that is, universal services for everybody with additional support for families with greater need (Marmot et al. 2010; Cowley, Whittaker, et al. 2018).

Health visitors are specialists in community public health regulated by the UK‐wide Nursing and Midwifery Council (NMC). They are a key health professional group dedicated to community health and the well‐being of babies and children under five years of age, and their parents or primary caregivers in the United Kingdom. Health visitors and the skill mix workers in their teams operate almost exclusively in homes or community settings. Their provision has been described as the ‘…backbone of early years services across the UK … the “safety net” around all families’ (UNICEF‐UK 2022, 21), reflecting the fact that health visitors provide a universal service, reaching the vast majority of infants born in the United Kingdom (Office for Health Improvement and Disparities 2024).

Health visitors focus on building strong and trusting relationships with children under five years of age and their families, to support and influence positive health outcomes in the immediate and longer term (Bidmead et al. 2015). They have a particular orientation to practice, which is underpinned by a social justice lens that involves them addressing the needs of not only the individual and family but the wider community; that is, both immediate health concerns and the wider determinants of poor health (Fanner et al. 2022).

However, health visiting services have changed significantly in terms of the funding context and demand since the turn of the century. Constitutional changes mean that Scottish, Welsh and Northern Ireland governments have devolved powers over health services, including health visiting. England has seen a more marked increase in the use of skill‐mix practitioners (such as community nursery nurses and community staff nurses) (Whittaker et al. 2021) to support the delivery of services to families, and significant variation in ‘service offer’ in terms of health visiting caseloads and practice (Reid and Tracey 2023; Institute of Health Visiting 2025).

Over the past decade, there has been a significant worsening in the social gradient of health in the United Kingdom (Marmot et al. 2010, 2020), with increasing numbers of children growing up in poverty (Department for Work and Pensions [DWP] 2023) and related increases in infant morbidity and mortality (Goldblatt 2024). There are higher numbers of children in need of child protection (Institute of Health Visiting 2025), and while the full impact is still being assessed, the COVID‐19 pandemic had a significantly adverse impact on both health visiting services and the families they serve (Morton and Adams 2022).

Nurturing care and the home environment are key to both preventing and protecting against social adversity (Britto et al. 2017), reflecting the way in which adversity is transmitted across generations through its biological embedding during sensitive developmental periods. For example, severe maternal stress during pregnancy and the first two years of life impacts the child's rapidly developing nervous system directly and indirectly, and, in particular, the hypothalamic‐pituitary‐adrenal axis, with long‐term implications for all aspects of the child's later development (Fox et al. 2010; Shonkoff 2012). The first 1001 days are a significant window of opportunity to give every child the best start in life, and to mitigate the impact of early childhood adversity. Health visitors are the lead practitioners with both the expertise and statutory responsibility to support families during this period.^1^ As such, health visitors have a key role to play in reducing social inequalities and improving child health.

This Evidence and Gap Map (EGM) aims to collate and map research evidence evaluating the practice of health visitors and their teams across the United Kingdom in the course of their work with babies and children under five years of age and their families. The focus of this review on the United Kingdom is due to the significant differences in the role of health visitors/public health nurses across countries, particularly in terms of their training, the context of service delivery, and role expectations. Therefore, evidence from comparable roles in other countries should be explored in a separate review.

The focus will be on assessing what is known about the benefits of health visiting services, experiences of the delivery and receipt of such services, and the facilitators and barriers to the uptake and delivery of effective health visiting services in the context of the policy changes since 2004. The year 2004 is significant in policy terms for two reasons. First, there were significant legislative changes on how the profession is regulated, including closure of the dedicated health visitor register, the introduction of Standards of Proficiency: Specialist Community Public Health Nurses (SCPHN) (Nursing and Midwifery Council [NMC] 2004) and the new SCPHN part of the NMC register. Second, the fourth edition of ‘Health for All Children’ (Hall and Elliman 2006), stated that a single universal home visit may suffice for some families, potentially influencing views about the scope of health visiting services.

#### 1.1.2 The UK Policy Context for Health Visiting

In the last decade, there have been significant changes in UK government administration and policy. Each nation of the UK has specific political and policy contexts with differing numbers of universal mandated reviews offered to babies and children under five years of age and their families, as shown in Table 1. In addition, each of the four countries in the UK is currently subject to a revised NMC code (Nursing and Midwifery Council [NMC] 2018) and updated Standards of Proficiency for Specialist Community Public Health Nurses (Nursing and Midwifery Council [NMC] 2022) that detail health visitor‐specific proficiencies.

**Table 1:** Policy variations across the United Kingdom.

UK country	Child public health/prevention programmes	Health visiting profession	Commissioning and service organisation
Wales	Healthy Child Wales Programme (HCWP) (GIG Cymru/NHS Wales and Llywodreath Cymru 2016) was introduced in 2016 and updated in 2024 (this update related to school‐aged children five to 16). It offers a proportionately universal programme of eight to nine health reviews for all under fives. In addition, the Flying Start programme was launched in 2006/7 (Llywodreath Cymru/Welsh Government 2017) (which is multi‐disciplinary, with access to health visiting, parental support, child‐care and speech and language therapy) covers around 25% of children under the age of four in the most deprived areas, with health visitors offering a more intensive schedule of contacts.	Health visitors are encouraged to assess need for additional support using ‘Family Resilience Assessment Instrument Tool’. HCWP (2024) specifies that a home visit will be offered for five of the programme contacts. These are to be carried out by health visitors in the home, with attendance at well baby clinics encouraged for the remaining contacts.	Health visiting is commissioned by Health Education and Improvement, Wales (HEIW). Health visitors are employed by NHS Health Boards in Wales. There are eight to nine contact points in the HCWP.
Scotland	In 2013, the Scottish Government undertook a scoping exercise of health visiting practice in Scotland. A refocused approach to health visiting was published by the Scottish Government in 2013. The changes took into account the changing policy landscape relating to the early years and children and families, and sought to ensure that workforce capability and capacity would be equipped to successfully deliver these policies. This led to the introduction of the Universal Health Visiting Pathway (UHVP) (Scottish Government 2015). It consists of 11 health visitor home visits to all families with children under five years: eight within the first year of life, with three child health reviews between 13 months and four years old. Additional support is also provided according to the level of need in line with a proportionate universalism approach, where the service is provided to all families, but more of the service is provided to those with a greater need.	UHVP was introduced as a major change project with additional education and resources to support health visitors. It was designed to capitalise on health visitors' practice and professional judgement. All support and planning for children and young people in Scotland should be underpinned by ‘Getting it right for every child (GIRFEC)’ – the national approach to improving the outcomes and supporting the well‐being of children and young people. One of the core components of GIRFEC is the offer of advice and support from a ‘named person’ who is there to provide a clear point of contact if a child, young person or family want information, advice or help. When a child requires support across multiple services, the named person is also often central in requesting assistance from those services. For families with children under five, health visitors carry out the ‘named person’ function.	Health visitors are employed by NHS Boards. The UHVP specifies that the 11 home visits are to be carried out by health visitors. There are 11 contact points in the UHVP.
Northern Ireland	Healthy Child, Healthy Future (HCHF) (Department of Health, Social Services and Public Safety 2010) is a framework for the Universal Child Health Promotion Programme in Northern Ireland, from pregnancy to 19 years, published by the Department of Health, Social Services and Public Safety in 2010. The current version is based on ‘Health for All Children’ fourth Edition, which is currently under refresh, so the next version will consider new evidence from ‘Health for All Children’ fifth Edition (Emond 2019) and other evidence‐based sources.	The current HCHF programme (2010) is grounded on the principles of progressive universalism based on the ‘Threshold of Needs Model’ (Department of Health, Social Services and Public Safety 2010). Some families will require only the minimum number of set contacts in level one, with additional services targeted, according to need, to those families in levels two to four. The nature of family life will mean that families will move in and out of the levels, and services will be adjusted accordingly.	Health visitors are employed by the five NHS Trusts in Northern Ireland. The current HCHF programme (2010) mandates a pre‐school Health Visiting service made up of nine core contacts, starting in the antenatal period. There are eight contact points in the HCHF.
England	The Healthy Child Programme (HCP) (Office for Health Improvement and Disparities 2023) was first introduced in 2009 and has been updated several times since. The most recent iteration (2023) integrated the 0–5‐year‐old part of the HCP with a wide range of other child public health programmes, such as Start for Life and Family Hubs, and with programmes for school‐aged children (who are usually served by school nurses). It promotes a continuum of services based in the community, ranging from universal to targeted and specialist provision.	The HCP (2023a) is designed to be delivered by a range of frontline workers, although guidance stresses that the team is to be led by health visitors for families with children under five years of age. These teams include a range of other workers, like community nursery nurses or community staff nurses, and a wide variety of different models exist for managing caseloads. There are no specifications about how the ‘health reviews’ are to be provided (e.g., at home, in a well baby clinic, through virtual contact), or which worker is to carry them out.	Health visiting services are commissioned by 317 local authorities (LAs), funded by the public health grant and following annual commissioning guidance issued by the Department of Health and Social Care. Most health visitors are employed within the NHS, but an increasing number are commissioned through charities or private providers, or employed directly by the LA. There are five contact points in the HCP.
UK‐wide	To become a health visitor, registered nurses or midwives must complete an approved postgraduate programme validated against the 2022 Standards of Proficiency for Specialist Community Public Health Nursing (SCPHN); they are regulated by the NMC. Skill mix teams often includes community staff nurses, who are first‐level registered nurses, from either adult, child, learning difficulties or mental health fields of practice. Community nursery nurses are unregulated workers who have usually completed a two‐year diploma in early years, but they are increasingly trained to foundation degree and degree level. Other community and family health staff may also be employed in health visiting teams with variable educational preparation, though this tends to be at Level two or three NVQ qualification or GCSEs (the qualifications achieved during compulsory education)		

There has also been a significant change in the workforce, in both the numbers of health visitors and health visiting skill‐mix practitioners, particularly in England. There has been a fall of more than 40% in the health visitor workforce (NHS Digital 2024) since the last national recruitment programme (Department of Health 2011) concluded in 2015 with the expansion of skill mix (i.e., in which health visitors lead teams that include staff who are not trained health visitors such as community nursery nurses and community staff nurses) working to meet population need.

In contrast, the devolved nations of the UK have had somewhat more stable policy environments and sustained funding, with less changes in health visitor workforce numbers. Specifically, there has been a smaller decline in the numbers of health visitors and slower increases in skill‐mix working in Scotland (Home et al. 2021) and Northern Ireland (Department of Health [DH] and Health and Social Care [HSC] 2017), and a slight increase in both health visitor and skill‐mix numbers in Wales (StatsWales 2024).

Each nation of the UK has specific political and policy contexts with differing numbers of universal mandated reviews offered to babies and children under five years of age and their families (see Table 1). Most notably, health visiting in England differs from Scotland, Wales and Northern Ireland in being the only health visiting service not to be wholly embedded in the NHS and having a significantly reduced number of visits. Specifically, England's health visiting service is funded through public health budgets paid directly to local government from the Department of Health and Social Care. This introduces an additional commissioning layer between national and local government policies. As a result, it is subject to considerable variation in local interpretation of national child health policy in England, for example, only 75 out of 150 eligible local authorities received Family Hubs and Start for Life Policy Programme (DHSC and DfE 2023) funding. It is perhaps also worth noting that while there is a variation in policy across the devolved nations, they have smaller populations and a closer relationship between funding and local decision‐making.

Each of the main policies informing universal health visiting provision draws upon the same evidence base about the health needs of babies, children aged under five years old and their parents, along with relevant research concerning advice, interventions or support for individual families. However, they provide very different guidance in terms of the nature and position of health visitors in the anticipated service and its constituent parts. In Scotland, for example, the ‘Universal Health Visitor Pathway’ (UHVP) specifies not only the number of contacts and anticipated needs to be reviewed and responded to, but that they are to be carried out by a health visitor, during a home visit. This implies that a particular contact is to be delivered through the judicial application of health visitors' *knowledge* (e.g., evidence of needs and appropriate response, understanding local area and the community as well as the wider population), *skills* (like needs assessment, relationship building, communication, cultural safety) and *approach to delivery* (specifically a home visit, not through clinic attendance or remote contact, for example). Each home visit includes age‐related assessments, advice, screening observations and tests, all specified in the pathway, but in short, the policy rests upon a view that each contact delivers ‘health visiting’ as an intervention, in the home.

In contrast, in England, the five mandated contacts also rest upon knowledge and evidence of anticipated health needs and appropriate responses at different ages, but there is no specification about who should carry them out or how – only that the person must be employed within a team led by a qualified health visitor. Despite naming health visitors in the mandate, this policy separates the contact (named a ‘health review’) from its application, and therefore from the knowledge and skills of the person undertaking the review. In England, as elsewhere, each health review includes specified age‐related assessments, advice, screening observations and tests, but essentially the contact itself (regardless of where and how it is carried out) is regarded as the intervention, since health visitors' professional skills and capabilities are largely discounted in this policy.

The above policy context will provide the backdrop against which the evidence identified by this review will be assessed, and the impact of differences in policy across the four nations will be examined in the discussion where it appears to be of relevance.

#### 1.1.3 The Intervention

This review focuses on all interventions undertaken as part of the delivery of health visiting services aimed at expectant parents, babies and children under five years of age and their families within the United Kingdom. This EGM will identify the empirical evidence, with regard to the impact of the core health visiting interventions, stakeholder experiences of delivering or receiving such interventions, and barriers/facilitators to their uptake or delivery. It should be noted that health visiting interventions may be delivered by health visitors or members of the health visiting team. This is addressed further in the description of the population below.

#### 1.1.4 Why It Is Important to Develop the EGM

There are now regular rapid reviews of the evidence to inform new versions of the mandated ‘Healthy Child Programme’ in England (Axford et al. 2015; Asmussen and Brims 2018), in addition to regular reviews to inform NICE guidance (relevant to England, Wales and Northern Ireland) and SIGN (relevant to Scotland) about issues related to the population of interest in this EGM, including for example postnatal care (NG 194, National Institute for Health and Social Care [NICE] 2021), maternal and child nutrition (NG247, National Institute for Health and Social Care [NICE] 2025) or oral health in early years settings (QS 139, National Institute for Health and Social Care [NICE] 2016; SIGN 2014, etc.). There is also a plethora of research about effective interventions to prevent and treat common health problems that occur in children under the age of five years, including, for example, those included in the most recent ‘Health for All Children’ (Emond 2019).

However, none of the above reviews focus explicitly on health visiting activities. The last review of evidence about health visiting encompassed 30 years of health visiting research up to 2012 (Cowley et al. 2015). That review demonstrated the potential for health visitors to support health improvements, particularly in the critical foundation years between conception and age five, and documented the wide range of activities and underpinning principles of health visiting. Therefore, there is now a need for a review that collates available evidence and identifies gaps in the research across the past two decades in terms of the benefits of health visiting, and the facilitators and barriers to effective health visiting practice, within the changed policy context in the United Kingdom.

Finally, commissioners and service managers need dependable information to enable them to make the best funding decisions and resourcing of health visiting services. An EGM provides a visual tool that enables knowledge users to readily locate relevant evidence. It also supports the prioritisation of future research by objectively identifying gaps in knowledge.

## 2 Objectives

The overarching objective of this EGM is to identify what is known and where the evidence gaps are, in terms of (a) the impact of health visiting interventions; (b) experiences of delivering or receiving health visiting services across the UK; and (c) the barriers and facilitators to uptake or delivery of health visiting services. The focus will be inclusive in terms of study design and what is being assessed by the study with regard to outcome (see below for further information).

## 3 Methods

### 3.1 EGM: Definition and Purpose

Research evidence is often scattered across different sources and repositories that are not always accessible to key stakeholders (Snilstveit et al. 2016). This, together with the ubiquitous nature of classical ‘narrowly‐focused’ systematic reviews (Bastian et al. 2010), and a general paucity of complete overviews of the existing evidence, can mean ineffective or limited use of the known evidence within a specific area of professional practice or policy (Snilstveit et al. 2016). To deal with this methodologically, a relatively novel evidence synthesis method called an EGM (White et al. 2020) will be used for the purpose of the proposed review. EGMs are similar to scoping reviews and evidence maps in their shared purpose to ascertain the ‘Big Picture’, and provide a ‘…systematic presentation of all relevant evidence of a specified kind for a particular sector … to a specific research question’ (Campbell et al. 2023, 5).

The purpose of the EGM is to show what is known and where the evidence gaps are, about health visiting activities and services, and their outcomes, in the United Kingdom.

### 3.2 Framework Development and Scope

The EGM framework and scope were developed by the authors who have different areas of professional, methodological and lay expertise and experiences, in addition to being generally guided by the current (and recent historical) policy frameworks in which UK health visiting operates. This framework informs the inclusion and exclusion criteria used in the review.

### 3.3 Stakeholder Engagement

Stakeholders have played a central role in defining the scope of this EGM through their involvement in the development of the framework and the development of research questions on which to focus. The key stakeholders for this EGM include health visiting and social science academics, who make up the core advisory group. The EGM protocol has also been drafted in collaboration with a lay person with relevant experience (a parent), who is part of the formal advisory group. H. C. is a parent of three children aged five and under, who has offered extremely valuable advice and suggestions on the scope and direction. Last, a research fellow (A. S. M.) will assist the lead author (M. F.) with the searching and selection of literature for the EGM. The formal Advisory Group will meet monthly throughout the course of the review.

### 3.4 Context for Health Visiting

**Figure 1:**
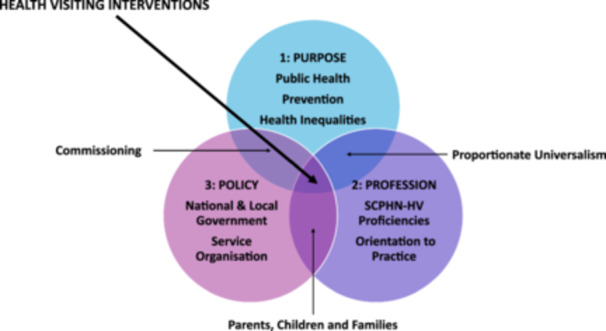
Context for Health Visiting.

The evidence used within health visiting policy and practice is diverse because it represents the needs of individuals, communities and populations. Furthermore, the evaluation of health visiting interventions is complicated by three different aspects of the context in which the services are delivered. These are illustrated in three overlapping spheres of (1) Purpose, (2) Profession and (3) Policy, shown in Figure 1 and explained below. These all converge upon and affect the central point; the interventions that are the focus of this EGM.

#### 3.4.1 Sphere 1 – Purpose

The purpose of health visiting is to improve public health, specifically by delivering diverse interventions focused on prevention, public health and reduction of health inequalities. Health visiting services are provided at both an individual and community level to improve public health through the delivery of a preventive service to the whole population of under five year olds and their families (Cowley, Whittaker, et al. 2018) in addition to the provision of a range of community‐based services. Proportionate universalism is defined in terms of the need for health interventions to be universal in offer and accessibility but delivered with an intensity and scale proportionate to the level of presenting (or potential) need (Marmot et al. 2010; Carey et al. 2015). Determining the level of intervention required, through assessment of family health needs, is a major element of health visiting practice (Cowley et al. 2015).

#### 3.4.2 Sphere 2 – Profession

Health visiting interventions are those delivered by practitioners qualified through the proficiencies for Specialist Community Public Health Nursing (health visiting) (SCPHN‐HV) (NMC 2022), or members of a team led by a health visitor. The impact of health visiting services is potentially related to health visitors' particular capabilities, gained through initial preparation, continuing professional development and supervision, although the distinctive nature of health visiting and health visiting services is under‐researched. Evidence from the ‘Why Health Visiting’ review integrated multiple different aspects into an explanatory framework described as an ‘orientation to practice’ (Cowley et al. 2015). This illustrates how a combination of the values, attitudes and attributes gained by health visitors through their initial preparation and later experience becomes embedded into their particular ‘way‐of‐being’ in practice (Malone et al. 2016). The orientation to practice describes how health visiting practice embodies collected knowledge, values and capabilities:

1. From social and psychological research about how to create health, known as ‘salutogenesis’,
2. In recognising each person (and family) in their own unique situations, taking account of a wider human and social ecology and
3. Involving a deep human valuing that underpins a need for an individualised, non‐judgemental and person‐centred service.

This orientation does not describe health visiting practice ‘as it is’, but provides a framework derived from an analysis of more than 30 years of research about the profession, which can provide a theoretical basis for linking future studies, and health visitor preparation, to wider social and psychological research.

#### 3.4.3 Sphere 3: Policy

Health visiting services are closely affected by the policy decisions, at both a national and local level. Significant differences in terms of the detail of guidance across the four UK countries and the extent to which specific aspects of provision are mandated or advised when organising services were highlighted above. By overseeing funds for the service, commissioners are the link between identified health needs to be addressed in their local area and nationally mandated policy. Harron et al. (2024) have explored how different approaches to these multiple requirements may affect health visiting practice, along with many other factors, including research about which approaches work best (or not), that need to be considered by service managers as they plan how best to operate the provision.

Each of these different aspects overlaps, as shown in Figure 1, providing the context for the particular, individual health visiting interventions received by parents, their babies and children, shown where the three spheres intersect. This context will be used to help us understand and explain the results of the evidence included in this review with regard to impact, experiences, and facilitators/barriers.

### 3.5 Dimensions

The primary dimensions for this review relate to health visitor interventions and outcomes in terms of three domains – impact, stakeholder experience, and facilitators/barriers to service uptake/delivery. Other secondary dimensions (segmenting filters) will include the study design, type of worker (e.g., Specialist Health Visitor, Health Visitor, Skill‐Mix Practitioners), country (United Kingdom, Scotland, Northern Ireland, Wales, England), location/situation (Home environment, Community setting, Remote ‐ Digital or Telephone). We will produce a range of tables based on these primary and secondary dimensions.

#### 3.5.1 Types of Study Design

We will include all types of study design (i.e., quantitative and qualitative) within both the published and grey literature. This reflects the fact that the review is addressing three research questions that will each involve the use of different types of evidence – for example, impact; quantitative; experiences: qualitative; facilitators and barriers: both quantitative and qualitative. We will also identify secondary research literature, such as systematic reviews that focus on one of the interventions defined below, and evidence from economic evaluations. We will include data from ongoing studies within the specified search dates.

#### 3.5.2 Types of Intervention

Health visitors (including specialist health visitors) and other health visiting team members deliver a range of interventions as part of their remit to promote the health and well‐being of families with children (conception to age five). While terminology for prevention, promotion and protection of health varies, for the purposes of this protocol, we use ‘primary’, ‘secondary’ and ‘tertiary’ to describe health visiting interventions (see Figure 1). Health visiting interventions span promotion in which they work on a population basis with all pregnant and new mothers/fathers to promote their wellbeing and prevent later problems through screening and health promotion activities on a universal basis (primary level); on a selective basis (secondary level) with, for example, women who have been identified as being at greater risk of poor outcomes (e.g., women screened and diagnosed with mild to moderate mental health problems for whom they might deliver as series of listening visits), and on a indicated basis (tertiary level), where there are significant mental health, substance use or interpersonal violence problems for example and the health visitor works with other services to help support the women and her family in the community.

In addition to working on a one‐to‐one basis with parent‐infant dyads/families, health visitors also work at a community level, with groups or communities in need of additional support.

Health visiting interventions at all of the above levels will be included in this review, and will be categorised based on the level at which they are delivered (e.g., English terms: universal; universal plus; universal partnership plus). In addition, health visiting interventions will also be categorised in relation to how they are:

- **Organised** (e.g., established programmes and models, multi‐agency, workforce recruitment and retention, practitioner wellbeing and support, caseload management, and key performance indicators/electronic health records);
- **Educated and trained** (e.g., initial preparation, continuous professional development/advanced practice, and specific knowledge and skills); and
- **Supervised** (e.g., safeguarding supervision, clinical supervision, mentorship/preceptorship, leadership, and skill mix teams).

#### 3.5.3 Types of Population

The population will be health visitors and other health visiting team members working with all expectant parents, babies and children under five years of age and their families as part of routine health visiting services, including universal, targeted and specialist provision in the UK. Health visiting team members include all clinical staff who are working under the supervision of health visitors (e.g., community staff nurses; community nursery nurses) to deliver their country‐specific child public health programme, for example, England's Healthy Child Programme.

#### 3.5.4 Types of Outcome Measures

An outcome may be the achievement of a process goal, delivery of a specified aspect of practice by a health visitor or member of the health visiting team, or research about facilitators and barriers to effective working within different organisations (or reorganisation) of services. We will include studies that identify and evaluate the impact of health visiting interventions, using data from quantitative, qualitative, and mixed methods research. Example outcomes for the three research questions are as follows (this list is not exhaustive):

- ***Intervention impact***
  - **Maternal –** this refers to all aspects of maternal functioning, including mental health, physical health, parenting, breastfeeding, and so forth.
  - **Paternal** – this refers to all aspects of paternal function, including mental health, physical health, parenting, health inequalities, and so forth.
  - **Whole Family**
  - **Child** – this includes all aspects of infant functioning, including feeding, child development, immunisation, oral health, and so forth.
  - **Caregiver‐child –** this includes all measures related to the relationship, including interaction, and other relationship measures
  - **Other Community** – this refers to all aspects of communities that engage with health visiting, beyond an individual level, such as community capacity and social cohesion, and so forth.
- ***Experiences of service delivery or receipt***
  - This includes key stakeholder experiences of either delivering or receiving any of the health visiting services referred to above, and that do not explicitly focus on facilitators and barriers (see next item).
- ***Facilitators and barriers to service uptake or delivery***
  - This includes all outcomes focused on factors that have been identified either quantitatively or qualitatively as facilitating or hindering the uptake, delivery or receipt of health visiting services, and so forth. For example, availability and accommodation, acceptability, accessibility, referral rates, collaborative activities and cultural safety, and so forth.

We will produce a glossary of terms for all outcomes included. Where clear measures are not available or possible, we will explore the potential of using ‘markers of success’ as a category of outcome, drawing on the examples provided by Cowley, Malone, et al. (2018) and Cowley, Whittaker, et al. (2018).

### 3.6 Other Eligibility Criteria

#### 3.6.1 Types of Location/Situation

Studies that have included health visitors or health visiting teams working within the UK will be eligible for inclusion.

#### 3.6.2 Types of Settings

Studies that include health visiting activities conducted within the home environment, community setting (including clinics), or remote setting (digital or telephone) will be eligible for inclusion.

### 3.7 Search Methods and Sources

**Databases and Years to Be Searched**: Searches for relevant evidence will be conducted using key electronic databases accessible through the University of Oxford Bodleian Library, including bibliographic providers such as ProQuest Social Science Premium, Scopus, Web of Science Core Collection, PubMed, Ovid for PsycINFO and EBSCO Host CINAHL, and the grey literature. The latter will include the following:

**King's Fund Library Database:** This database is a valuable source for health and social care policy and management, including grey literature such as research reports from trusts, charities, and government agencies.

**HMIC (Health Management Information Centre):** HMIC covers journal articles, official publications, books, reports, and pamphlets relevant to health management and policy, making it a comprehensive resource.

**Cochrane Library:** This library contains various databases that provide independent, high‐quality evidence to inform healthcare decisions, including grey literature related to health interventions.

**Other useful resources:** OpenMD is a search engine for health information, and OpenDOAR provides access to open‐access repositories worldwide. Additionally, ClinicalTrials.gov is a registry of clinical trials.

**Proquest Dissertation and Theses:** covers all published dissertations and theses from Master's through Doctoral‐level research.

We will also search within publication lists of known health visiting academics and research centres for relevant literature (e.g., Institute of Health Visiting). We will also hand search specific journals akin to health visiting, including Community Practitioner and the Journal of Health Visiting (now Journal of Family and Child Health).

This EGM will capture all relevant research literature from 2004, the year that marked a major change in health visitor preparation, up until the end of June 2025. This will also capture most research publications stemming from New Labour's major early years policy reforms, which indirectly affected health visiting practice, including the piloting and expansion of the Sure Start Local Programmes Policy (1999–2003), evolution of Sure Start Children's Centres (2003–2010) and the implementation of Family Nurse Partnership programmes through the Coalition and Conservative governments (2010–2024) with the introduction of Family Hubs and Start for Life Programmes. It will encompass evaluations of significant reforms in Scotland, including the Universal Health Visiting Pathway (Scottish Government 2015) and Wales, including the Flying Start programme (Welsh Government 2017).

**Search terms:** The search terms to be used will focus on optimising the sensitivity of the search to ensure inclusivity and will be adapted as appropriate for the different databases.

We will focus on identifying relevant articles using a range of *intervention* (e.g., health visit* or community nurse or nursery nurse, etc.) combined with population (e.g., 0–5; pregnan*; preschool; antenatal; prenatal; postnatal; birth visit; expectant parent*; expectant mother, etc.) terms anywhere in the paper, in the first instance. To increase the specificity, we might also limit the fields in which these terms can appear. We might also include some study design terms (e.g., qual* or quant* or review or RCT or quasi scientific; or evaluation, etc.).

The following is the final set of search terms being used for CINAHL (which is one of the key databases for health visiting publications) in two separate searches:

*Search 1*

- AB (health visit* or specialist community public health nurs* or SCPHN) OR MH (health visit* or specialist community public health nurs* or SCPHN);
- Publication Date: 2004/01/01–2025/06/30; Peer Reviewed; Geographic Subset: UK & Ireland; Location of Practice: United Kingdom; Language: English;
- Expanders – Apply related words; Apply equivalent subjects;
- Search modes – Proximity.

*Search 2*

1. AB (0–5; pregnan*; preschool; antenatal; prenatal; postnatal; birth visit; expectant parent*; expectant mother) OR MH (0–5; pregnan*; preschool; antenatal; prenatal; postnatal; birth visit; expectant parent*; expectant mother) AND AB (home visit* or MESCH or Maternal Early Childhood Sustained Home‐visiting or FNP or Family Nurse Partnership or Family Partnership) OR MH (home visit* or MESCH or Maternal Early Childhood Sustained Home‐visiting or FNP or Family Nurse Partnership or Family Partnership);
2. Publication Date: 2004/01/01–2025/06/30; Peer Reviewed; Geographic Subset: UK & Ireland; Location of Practice: United Kingdom; Language: English;
3. Expanders – Apply related words; Apply equivalent subjects;
4. Search modes – Proximity.

### 3.8 Analysis and Presentation

#### 3.8.1 Report Structure

The EGM will be presented as an online interactive EGM as well as a narrative review paper for publication.

#### 3.8.2 Filters for Presentation

In the online interactive map, additional dimensions (e.g., country; delivery context) will enable users to select studies meeting those criteria. Study design will be used as a segmenting filter. In the hard copy, multiple 2 × 2 representations of the EGM will be reported using the above filters. We will provide detailed definitions for each additional dimension and describe how the different dimensions will be captured in the map.

#### 3.8.3 Dependency

Original studies will be our primary unit of analysis, but follow‐up studies and analyses of different sets of variables will be treated as the same.

### 3.9 Data Collection and Analysis

#### 3.9.1 Screening and Study Selection

Screening of papers will be undertaken in two stages:

Stage one: Two review authors (M. F. and A. S. M.) will screen the titles and abstracts of studies identified by the searches to assess whether they meet the inclusion criteria. All eligible studies and those about which there is uncertainty will be included in the next stage of screening.

Stage two: a full paper blind review will be undertaken of all remaining papers by two of three reviewers (M. F., A. S. M. and J. B.). Discrepancies between reviewers will be resolved by discussion with a third and fourth review author (S. C. and K. W.).

#### 3.9.2 Data Extraction and Management

One review author (A. S. M.) will extract data using a specially designed data extraction form and will enter the data into EPPI‐Reviewer software (Thomas et al. 2023). This data extraction will blind checked by one other review author (M. F. or S. C.). Differences in interpretation will be reconciled through discussions with the author team.

Where data are not available in the published study reports, we will contact study investigators to request missing information.

We will extract the following information from each of the included studies: study design; location/situation for delivery of service; level of prevention; types of intervention; type of service organisation; population; type of outcome; outcome data. Data extraction from systematic reviews will follow a similar format but will additionally involve the extraction of data relating to the number of studies included, critical appraisal, and method of data synthesis.

#### 3.9.3 Tools for Assessing Risk of Bias/Study Quality of Included Reviews

No appraisal of study quality or risk of bias is planned, due to the diverse study designs being included. We will be using the study design as a filtering segment to illustrate the range of research methods used within health visiting research evidence.

#### 3.9.4 Methods for Mapping

EPPI‐Mapper (Digital Solution Foundry and EPPI Centre 2023) will be used to assist with the mapping of this EGM, with support from F. C. (co‐author). The mapping will be conducted independently by two authors (M. F. and A. S. M.), and reconciliation will take place where differences arise through discussion with the author team.

## Author Contributions

The authors bring a wide range of distinct expertise and experiences to this EGM in terms of direct health visiting practice, evidence‐based policy formation and evaluation, research methodologies and lived experience. The whole team will contribute to the contents of the EGM in both the drafting and the final mapping. Jane Barlow and Fiona Campbell bring expertise and experience to EGM methods. Michael Fanner, Sarah Cowley, Karen Whittaker, Fiona Campbell and Mary Malone are qualified health visitors. Helen Critcher provides a parent/caregiver perspective to the EGM.

Michael Fanner and Anita Schrader McMillan will be responsible for the searching and retrieval of the information required for the EGM. We will seek statistical analysis expertise as and when required from a statistical expert within the Department of Social Policy and Intervention, University of Oxford.

## Conflicts of Interest

Michael Fanner was involved as a co‐investigator on one study during the review period (since 2004) that was either directly concerned with health visiting practice or involved health visitors as one group of the practitioners delivering an intervention. Until September 2025, Michael Fanner was a Trustee for the Institute of Health Visiting (iHV), a charity and professional body that will have an interest in the results and conclusions of the review.

Jane Barlow was involved as a principal or co‐investigator on four studies during the review period (since 2004) that were either directly concerned with health visiting practice or involved health visitors as one group of the practitioners delivering an intervention.

Sarah Cowley was involved as a principal or co‐investigator on eight studies during the review period (since 2004) that were either directly concerned with health visiting practice or involved health visitors as one group of the practitioners delivering an intervention. Sarah Cowley has provided supervision to various doctoral and Master's students whose published work may be eligible for review. Sarah Cowley is a patron for ‘MESCH‐UK’, an Australian maternal and early childhood sustained home visiting programme being implemented across the United Kingdom. Sarah Cowley is also a Trustee for the iHV, a charity and professional body for health visiting, which will have an interest in the results and conclusions of the review.

Karen Whittaker is employed by the iHV, a charity and professional body for health visiting, which has a vested interest in the conclusions of the review, as the information provided will be relevant to future iHV priorities. Karen Whittaker was involved in as either a lead investigator or collaborator with six studies during the review period (since 2004) that were either directly concerned with health visiting practice or involved health visitors as one group of the practitioners delivering an intervention. Karen Whittaker has 10 publications from these studies that may or may not be included within the review.

Mary Malone is a professor in the Florence Nightingale Faculty of Nursing and Midwifery, which has a vested interest in the conclusions of the review as the information provided will be relevant to future educational opportunities for health visitors, nurses and midwives. Mary Malone was involved in as either a lead investigator or collaborator with four studies and 14 papers during the review period (since 2004) that were either directly concerned with health visiting practice or involved health visitors as one group of the practitioners delivering an intervention.

The other authors declare no conflicts of interest.

## Plans for Updating the EGM

We plan to update the map when sufficient further studies and resources become available.

## Differences Between Protocol and Full Report

The authors have nothing to report.

## Sources of Support

**Internal Sources**

- No sources of support provided.

**External Sources**

- No sources of support provided.

## Supporting information

Framework_for_EGM.

## Data Availability

The authors have nothing to report.

## Endnotes

1 This statutory responsibility is outlined in Section 2 within Section 12 of the UK's Health and Social Care Act 2012.

## References

[cl270078-bib-0001] Asmussen, K. , and L. Brims . 2018. What Works to Enhance the Effectiveness of the Healthy Child Programme: An Evidence Update. Early Intervention Foundation. https://www.eif.org.uk/report/what‐works‐to‐enhance‐the‐effectiveness‐of‐the‐healthy‐child‐programme‐an‐evidence‐update.

[cl270078-bib-0002] Axford, N. , J. Barlow , J. Coad , et al. 2015. Rapid Review of Evidence to Update the Healthy Child Programme. Public Health England. https://assets.publishing.service.gov.uk/media/5a74fd6540f0b6399b2afc9e/150520RapidReviewHealthyChildProg_UPDATE_poisons_final.pdf.

[cl270078-bib-0003] Bastian, H. , P. Glasziou , and I. Chalmers . 2010. “Seventy‐Five Trials and Eleven Systematic Reviews a day: How Will we Ever Keep up?” PLoS Medicine 7, no. 9: e1000326. 20877712 10.1371/journal.pmed.1000326 PMC2943439

[cl270078-bib-0059] Bidmead, C. , S. Cowley , and P. Grocott . 2015. “Investigating the Parent/Health Visitor Relationship: Can it be Measured?” Journal of Health Visiting 3, no. 10: 548–557.

[cl270078-bib-0005] Britto, P. R. , S. J. Lye , K. Proulx , et al. 2017. “Nurturing Care: Promoting Early Childhood Development.” Lancet 389, no. 10064: 91–102. 10.1016/s0140-6736(16)31390-3. 27717615

[cl270078-bib-0007] Campbell, F. , A. C. Tricco , Z. Munn , et al. 2023. “Mapping Reviews, Scoping Reviews, and Evidence and Gap Maps (EGMs): The Same but Different—The “Big Picture” Review Family.” Systematic Reviews 12, no. 1: 45. 10.1186/s13643-023-02178-5. 36918977 PMC10014395

[cl270078-bib-0060] Carey, G. , B. Crammond , and E. De Leeuw . 2015. “Towards Health Equity: A Framework for the Application of Proportionate Universalism.” International Journal for Equity in Health 14: 81. 10.1186/s12939-015-0207-6. 26369339 PMC4570091

[cl270078-bib-0010] Cowley, S. , K. Whittaker , M. Malone , S. Donetto , A. Grigulis , and J. Maben . 2015. “Why Health Visiting? Examining the Potential Public Health Benefits From Health Visiting Practice Within a Universal Service: A Narrative Review of the Literature.” International Journal of Nursing Studies 52: 465–480. 10.1016/j.ijnurstu.2014.07.013. 25304286

[cl270078-bib-0011] Cowley, S. , K. Whittaker , M. Malone , S. Donetto , A. Grigulis , and J. Maben . 2018. “What Makes Health Visiting Successful—Or Not? 1. Universality.” Journal of Health Visiting 6, no. 7: 352–360.

[cl270078-bib-0009] Cowley, S. , M. Malone , K. Whittaker , S. Donetto , A. Grigulis , and J. Maben . 2018. “What Makes Health Visiting Successful—Or Not? 2. The Service Journey.” Journal of Health Visiting 6, no. 8: 404–412.

[cl270078-bib-0012] Department for Work and Pensions (DWP) . 2023. Household Below Average Income Statistics. Accessed February 20, 2024. https://www.gov.uk/government/collections/households‐below‐average‐income‐hbai‐‐2.

[cl270078-bib-0016] Department of Health (DH) and Health and Social Care (HSC) . 2017. Delivering Care Phase 4 Health Visiting. A Policy Framework for Nursing and Midwifery Workforce Planning in Northern Ireland. Accessed April 27, 2024. https://www.publichealth.hscni.net/sites/default/files/4.Delivering%20Care%20Summary%20Paper_Phase%204%20HV%20130117.pdf.

[cl270078-bib-0017] Department of Health, Social Services and Public Safety . 2010. Healthy Child, Healthy Future. A Framework for the Universal Child Health Promotion Programme in Northern Ireland. Accessed February 20, 2024. https://www.health‐ni.gov.uk/sites/default/files/publications/dhssps/healthychildhealthyfuture.pdf.

[cl270078-bib-0015] Department of Health . 2011. Health Visitor Implementation Plan 2011– 2015: A Call to Action. Department of Health. Accessed February 20, 2024. https://www.gov.uk/government/publications/health‐visitor‐implementation‐plan‐2011‐to‐2015.

[cl270078-bib-0062] Departmentof Health and Social Care (DHSC) and Department for Education (DfE) . 2023. Startfor Life and Family Hubs Programme. Accessed April 2, 2024. https://www.gov.uk/government/collections/family‐hubs‐and‐start‐for‐life‐programme.

[cl270078-bib-0019] Digital Solution Foundry and EPPI Centre . 2023. EPPI‐Mapper, Version 2.2.4. EPPI Centre, UCL Social Research Institute, University College London.

[cl270078-bib-0020] Dingwall, R. W. J. 1977. “Collectivism, Regionalism and Feminism: Health Visiting and British Social Policy 1850‐1975.” Journal of Social Policy 6, no. 3: 291–315.

[cl270078-bib-0021] Emond, A. , ed. 2019. Health for All Children (5th ed.). Oxford University Press.

[cl270078-bib-0022] Fanner, M. , K. Whittaker , and S. A. Cowley . 2022. “Being Orientated Towards Social Justice: Learning for Health Visitor Practice.” Nurse Education Today 116: 105386. 35849961 10.1016/j.nedt.2022.105386

[cl270078-bib-0024] Fox, S. E. , P. Levitt , and C. A. Nelson, III . 2010. “How the Timing and Quality of Early Experiences Influence the Development of Brain Architecture.” Child Development 81: 28–40. 10.1111/j.1467-8624.2009.01380.x. 20331653 PMC2846084

[cl270078-bib-0025] GIG Cymru/NHS Wales and Llywodreath Cymru . 2016. An Overview of the Healthy Child Wales Programme. Accessed April 26, 2024. https://www.gov.wales/sites/default/files/publications/2022‐03/an‐overview‐of‐the‐healthy‐child‐wales‐programme.pdf.

[cl270078-bib-0026] Goldblatt, P. 2024. Health Inequalities: Lives Cut Short. UCL Institute of Health Equity. Accessed February 20, 2024. https://www.instituteofhealthequity.org/resources‐reports/health‐inequalities‐lives‐cut‐short.

[cl270078-bib-0027] Hall, D. M. B. , and D. Elliman . 2006. Health for All Children (4th ed.). Oxford Medical Publications.

[cl270078-bib-0061] Harron, K. , S. Kendall , C. Bunting , et al. 2024. “How Should Policymakers, Funders, and Research Teams Mobilize to Build the Evidence Base on Universal Early Years Services?” Primary Health Care Research & Development 25: e67. 10.1017/S1463423624000550. 39655521 PMC11669806

[cl270078-bib-0028] Home, M. A. , L. Marryat , and R. Wood . 2021. Universal Health Visiting Pathway Evaluation – Phase 1: Report – Routine Data Analysis – Workforce. Accessed April 27, 2024. https://discovery.dundee.ac.uk/ws/portalfiles/portal/70863473/evaluation_universal_health_visiting_pathway_scotland_phase_1_report_routine_data_analysis_workforce.pdf.

[cl270078-bib-0029] Institute of Health Visiting . 2025. State of Health Visiting, UK Survey Report: From Disparity to Opportunity: The Case for Rebuilding Health Visiting. Accessed February 27, 2024. https://bit.ly/4hmR3Me.

[cl270078-bib-0030] Llywodreath Cymru/Welsh Government . 2017. *Flying Start Health Programme Guidance*. Accessed February 20, 2024. https://www.gov.wales/sites/default/files/publications/2019‐07/flying‐start‐health‐programme‐guidance_0.pdf.

[cl270078-bib-0031] Malone, M. , K. A. Whittaker , S. Cowley , I. Ezhova , and J. Maben . 2016. “Health Visitor Education for Today's Britain: Messages From a Narrative Review of the Health Visitor Literature.” Nurse Education Today 44: 175–186. 27429349 10.1016/j.nedt.2016.04.007

[cl270078-bib-0032] Marmot, M. , J. Allen , T. Boyce , P. Goldblatt , and J. Morrison . 2020. Health Equity in England: The Marmot Review 10 Years On. Institute of Health Equity. https://bit.ly/48vsR6i. 10.1136/bmj.m693 32094110

[cl270078-bib-0033] Marmot, M. , P. Goldblatt , J. Allen , et al. 2010. Fair Society, Healthy Lives (The Marmot Review). Institute of Health Equity. Accessed January 20, 2024. https://www.instituteofhealthequity.org/resources‐reports/fair‐society‐healthy‐lives‐the‐marmot‐review.

[cl270078-bib-0035] Morton, A. , and C. Adams . 2022. “Health Visiting in England: The Impact of the COVID‐19 Pandemic.” Public Health Nursing 39: 820–830. 10.1111/phn.13053. 35099079

[cl270078-bib-0036] National Institute for Health and Social Care (NICE) . 2016. Oral Health Promotion in the Community: Quality Statement 2: Early Years Settings and Schools QS139. NICE. Accessed January 10, 2024. https://www.nice.org.uk/guidance/qs139/chapter/quality‐statement‐2‐early‐years‐settings‐and‐schools.

[cl270078-bib-0037] National Institute for Health and Social Care (NICE) . 2021. NG 194. Postnatal Care: Breastfeeding Interventions. NG 194. NICE. Accessed January 10, 2024. https://www.nice.org.uk/guidance/ng194/evidence/p‐breastfeeding‐interventions‐pdf‐326764485980.

[cl270078-bib-0038] National Institute for Health and Social Care (NICE) . 2025. Maternal and Child Nutrition: Nutrition and Weight Management in Pregnancy, and Nutrition in Children Up to 5 Years. NG 247 NICE. Accessed January 24, 2025. https://www.nice.org.uk/guidance/ng247. 39999244

[cl270078-bib-0039] NHS Digital . 2024. *NHS Hospital & Community Health Service (HCHS) Monthly Workforce Statistics October 2023*. https://digital.nhs.uk/data‐and‐information/publications/statistical/nhs‐workforce‐statistics/october‐2023.

[cl270078-bib-0040] Nursing and Midwifery Council (NMC) . 2004. Standards of Proficiency: Specialist Community Public Health Nurses. Accessed April 27, 2025. https://www.nmc.org.uk/globalassets/sitedocuments/standards/nmc‐stand‐alone‐standards‐of‐proficiency‐for‐specialist‐community‐public‐health‐nurses.pdf.

[cl270078-bib-0041] Nursing and Midwifery Council (NMC) . 2018. *The Code*. Accessed December 18, 2023. https://www.nmc.org.uk/standards/code/.

[cl270078-bib-0042] Nursing and Midwifery Council (NMC) . 2022. *Standards of Proficiency for Specialist Community Public Health Nurses*. Accessed December 18, 2023. https://www.nmc.org.uk/standards/standards‐for‐post‐registration/standards‐of‐proficiency‐for‐specialist‐community‐public‐health‐nurses2/.

[cl270078-bib-0044] Office for Health Improvement and Disparities . 2023. *Healthy Child Programme: Schedule of Interventions*. Accessed April 25, 2024. https://www.gov.uk/government/publications/healthy‐child‐programme‐schedule‐of‐interventions.

[cl270078-bib-0045] Office for Health Improvement and Disparities . 2024. Health Visitor Service Delivery Metrics: Quarterly Data for 2023 to 2024. Accessed April 15, 2024. https://www.gov.uk/government/statistics/health‐visitor‐service‐delivery‐metrics‐quarterly‐data‐for‐2023‐to‐2024.

[cl270078-bib-0048] Reid, B. , and J. Tracey . 2023. “Health Visitor Workload: An Integrative Review of the Literature.” Primary Health Care 35: e1804. 10.7748/phc.2023.e1804.

[cl270078-bib-0050] Scottish Government . 2015. *Universal Health Visiting Pathway in Scotland: Pre‐Birth to Pre‐School*. Accessed February 20, 2024. https://www.gov.scot/publications/universal‐health‐visiting‐pathway‐scotland‐pre‐birth‐pre‐school/.

[cl270078-bib-0051] Shonkoff, J. 2012. “Leveraging the Biology of Adversity to Address the Roots of Disparities in Health and Wellbeing.” Proceedings of the National Academy of Sciences of the United States of America 109, no. Suppl 2: 17302–17307. 10.1073/pnas.1121259109. 23045654 PMC3477384

[cl270078-bib-0052] SIGN . 2014. Dental Interventions to Prevent Caries in Children. Accessed January 10, 2024. https://www.sign.ac.uk/our‐guidelines/dental‐interventions‐to‐prevent‐caries‐in‐children/.

[cl270078-bib-0053] Snilstveit, B. , M. Vojtkova , A. Bhavsar , J. Stevenson , and M. Gaarder . 2016. “Evidence and Gap Maps: A Tool for Promoting Evidence Informed Policy and Strategic Research Agendas.” Journal of Clinical Epidemiology 79: 120–129. 10.1016/j.jclinepi.2016.05.015. 27387966

[cl270078-bib-0054] StatsWales . 2024. Number of Full‐Time Equivalent (FTE) Health Visitors in the Flying Start Workforce, by Local Authority. Accessed April 27, 2024. https://statswales.gov.wales/Catalogue/Health‐and‐Social‐Care/NHS‐Primary‐and‐Community‐Activity/flying‐start/NumberoffulltimeequivalenthealthvisitorsintheFlyingStartworkforce‐by‐localauthority.

[cl270078-bib-0055] Thomas, J. , S. Graziosi , J. Brunton , et al. 2023. EPPI‐Reviewer: Advanced Software for Systematic Reviews, Maps and Evidence Synthesis. EPPI Centre, UCL Social Research Institute, University College London.

[cl270078-bib-0056] UNICEF‐UK . 2022. *Early Moments Matter: Guaranteeing the Best Start in Life for Every Baby and Toddler in England*. Accessed January 10, 2024. https://www.unicef.org.uk/wp‐content/uploads/2022/10/EarlyMomentsMatter_UNICEFUK_2022_PolicyReport.pdf.

[cl270078-bib-0057] White, H. , B. Albers , M. Gaarder , et al. 2020. “Guidance for Producing a Campbell Evidence and Gap Map.” Campbell Systematic Reviews 16: e1125. 10.1002/cl2.1125. 37016607 PMC8356343

[cl270078-bib-0058] Whittaker, K. , J. V. Appleton , S. Peckover , and C. Adams . 2021. “Organising Health Visiting Services in the UK: Frontline Perspectives.” Journal of Health Visiting 9, no. 2: 68–75.

